# Gene signatures from scRNA‐seq accurately quantify mast cells in biopsies in asthma

**DOI:** 10.1111/cea.13732

**Published:** 2020-09-23

**Authors:** Jian Jiang, Alen Faiz, Marijn Berg, Orestes A. Carpaij, Corneel J. Vermeulen, Sharon Brouwer, Laura Hesse, Sarah A. Teichmann, Nick H. T. ten Hacken, Wim Timens, Maarten van den Berge, Martijin C. Nawijn

**Affiliations:** ^1^ Groningen Research Institute for Asthma and COPD (GRIAC) University of Groningen Groningen The Netherlands; ^2^ Department of Pathology and Medical Biology University Medical Center Groningen University of Groningen Groningen The Netherlands; ^3^ Department of Pulmonology University Medical Center Groningen University of Groningen Groningen The Netherlands; ^4^ Respiratory Bioinformatics and Molecular Biology (RBMB) Faculty of Science University of Technology Sydney Ultimo NSW Australia; ^5^ Wellcome Sanger Institute Wellcome Genome Campus Cambridge UK; ^6^ Open Targets Wellcome Genome Campus Cambridge UK; ^7^ Theory of Condensed Matter Group Cavendish Laboratory/Dept Physics University of Cambridge Cambridge UK

To the Editor:

Respiratory disease, characterized by changes in the cells of the lung, can affect molecular phenotype of cells and the intercellular interactions, resulting in a disbalance in the relative proportions of individual cell types. Understanding these changes is essential to understand the pathophysiology of lung disease. Conventional “bulk” RNA sequencing (RNA‐seq), analysing the entire transcriptome of the tissue sample, provides information about average expression levels of each gene in the mixed cell population, whereas it does not consider the cellular heterogeneity in samples composed of more than one cell type.^1^ Single‐cell RNA‐seq (scRNA‐seq) assesses the transcriptome of a complex biological sample with single‐cell resolution, allowing identification of the relative frequency of discrete cell types and analysis of their transcriptomes.^1^ Nevertheless, analysing the transcriptomic signature in large numbers of patients by scRNA‐Seq is currently limited by its high costs. Mast cells are key regulatory cells driving the inflammatory process in asthma.^2^ Since they can be quantified by immunohistochemical staining for validation purposes, we used mast cells as an example of a rare cell population to assess the validity of our deconvolution approach. Recently, a number of bulk RNA‐seq deconvolution methods have become available,^3^ for instance of two deconvolution methods, namely support vector regression (SVR),^4^ the machine‐learning method implemented in CIBERSORT and non‐negative least square (NNLS),^5^ using a matrix of cell type selective genes identified with AutoGeneSc.^6^ Both approaches are designed to estimate relative proportion of the main, common cell types present in the sample. When we used these methods to estimate the number of mast cells, we found a poor correlation with the number of mast cells stained by immunohistochemistry in the biopsies, suggesting the CIBERSORT and NNLS are less reliable in the case of rare cell types. We explored the possibility to use scRNA‐Seq data from small numbers of subjects to specifically interrogate the relative cell type frequency of a rare cell population in a bulk RNA‐Seq data set obtained from a large asthma cohort.

We analysed the scRNA‐Seq data^7^ of bronchial biopsies from 6 asthma patients and 6 matched healthy controls. scRNA‐Seq data can be accessed at European Genome‐phenome Archive (EGA) (accession number: EGAS00001002649). Unsupervised clustering of the 27 213 cells obtained from the 12 subjects identified 11 clusters, including those containing epithelial cells (92.19%), endothelial cells (2.14%), fibroblasts (0.59%), smooth muscle cells (0.22%) and the cluster comprising multiple lymphocyte subsets (4.86%). Mast cells were the smallest cluster (0.20%). The healthy subjects contained few or no mast cells (0.026% of all cells) in their biopsies, while the abundance of mast cells was clearly increased in the asthma patients (0.176%).^7^ In the cluster of mast cells, the relative expression levels of *TPSB2*, *TPSAB1*, *PTGS2* and *HPGDS* were significantly higher (Top 50 highly expressed genes) than their mean expressions in any other clusters.^7^

To identify a gene signature that was specific and selective for mast cells in a mixed tissue sample, we selected only those genes that met the following criteria in the scRNA‐Seq data: (a) >70% of all reads of the gene measured in the total scRNA‐Seq data set mapped to the mast cell cluster; (b) the minimum cumulative number of reads expressed by all cells in the mast cell cluster >100; and (c) the minimum number of reads expressed by each cell in the mast cell cluster that expresses the gene ≥2, but dropouts (non‐expressing cells) are allowed. Because mast cells were nearly absent in healthy subjects, we only used the scRNA‐Seq data from the 6 asthma patients to generate the mast cell‐specific gene list. The criteria resulted in 11 mast cell‐specific genes: *TPSB2, TPSAB1, TPSD1, TESPA1, RGS13, SLC18A2, CPA3, MS4A2, HPGDS, ADCYAP1* and *HDC*, with, respectively, 84.8%, 83.5%, 79.3%, 78.7%, 78.2%, 74.6%, 74.5%, 73.4%, 72.7%, 71.3% and 70.8% of reads mapping to the mast cell cluster. *TPSB2, TPSAB1, TPSD1* and *CPA3* encode the well‐known mast cell proteases.^8^, ^9^ *MS4A2, HPGDS* and *HDC* were also reported as genes encoding well‐defined mast cell markers.^10^ Microarray‐based genome‐wide expression patterns of airway epithelial brushings showed that *TPSB2, TPSAB1* and *CPA3* (ranked 4‐6) were among the most differentially expressed genes associated with asthma.^2^ Human mast cells produce three soluble forms of tryptases: α (*TPSAB1*)‐, β (*TPSB2*)‐ and δ‐tryptase (*TPSD1*).^8^ Particularly, anti‐TPSAB1 antibody serves as a marker (AA1+) for indicating the presence and activity of mast cells.^11^ There are two mast cell phenotypes, one expressing high levels of tryptase but little or no chymase and one expressing tryptase, chymase and CPA3.^11^, ^12^ Although the expression of *CMA1* (chymase‐coding gene) was not detected in our scRNA‐Seq data, *TPSB2, TPSAB1, TPSD1* and *CPA3* were all identified as the mast cell‐specific genes, suggesting the genes selected by our approach likely represent both mast cell subtypes.

To test whether the mast cell gene signature could quantify their abundance in bulk RNA‐Seq data, we used a data set of bronchial biopsies from 69 asthma patients either on (n = 44) or off (n = 25) inhaled corticosteroid (ICS) treatment^13^ (Table 1). Sample collection, RNA extraction, library preparation, sequencing and data analysis were processed as described.^14^ Bulk RNA‐seq data have been deposited at the EGA (accession number: EGAS00001003735). Since we intended to study the genome‐wide expression of rare cells from a population of cells, we did not exclude lowly expressed genes. In total, 55 269 unique Ensembl gene IDs (mapping to 1597 unique human genes) were available for analyses.

**Table 1:** Patient characteristics

Characteristic	Persistent asthma with ICS	Persistent asthma without ICS
Number	44	25
PC20AMP (mg/mL) median [min, max]	59.9 [0.002, 640.0]	45.4 [0.02, 640.0]
FEV1 (%pred) mean [SD]	84.6 [18.12]	83.2 [12.43]
ICS dose (µg/d) median[min, max]	800 [28, 2000]	NA
Beta‐agonist use (n(%))	40 (91%)	13 (52%)
Sex (m/f)	19/25	14/11
Age (y) mean [SD]	47.7 [12.45]	45.4 [12.50]
FEV1/FVC (%) mean [SD]	70.2 [10.31]	70.2 [8.24]
Reversibility FEV1 (%) mean [SD]	9.2 [6.58]	9.6 [6.14]
Atopy (phadiatop) (n(%))	10 (76)	5 (78)
Current smoking status (Yes, No)	6/38	9/16
Blood eosinophils (*10^9/L) median [min, max]	0.18 [0.04, 0.78]	0.25 [0, 0.9]

Note

Asthma patients included in the study were defined by same criteria as described.^13^

Abbreviations: FEV1, forced expiratory volume at timed intervals of 1.0; FVC, forced vital capacity.

We calculated the sum of the log_2_‐transformed fragments per kilobase of transcript per million fragments mapped (FPKM) values of the 11 genes for each patient and used the composite value as the mast cell gene score. Then, we correlated the mast cell gene score to mast cell numbers determined by immunohistochemical staining of TPSAB1 (AA1) in matched biopsies. In the 69 asthma patients, the mast cell‐specific 11‐gene signature was significantly correlated with mast cell numbers in biopsies (Pearson, *r* = 0.42, *P* < .001, FDR = 0.0084) (Figure 1A), although more stringent selection of mast cell‐specific genes (>75% and >80% of reads mapped to the mast cell cluster, resulting in 5‐gene and 2‐gene signatures, respectively) worked equally well. In the 44 asthma patients taking ICS, the average mast cell gene scores were significantly lower (median = 4.45 ± 1.13) than those found in the 25 asthma patients without ICS usage (median = 5.12 ± 0.78 and *P* = .013, Mann‐Whitney test) (Figure 1B). Interestingly, the AA1 staining revealed only a trend towards decreased numbers of mast cells in the biopsies of ICS‐treated asthma patients (median = 6.11 ± 5.15) compared to the patients without taking ICS (median = 10.83 ± 7.85, *P* = .087, Mann‐Whitney test) (Figure 1C). These were consistent with the findings that ICS treatment reduced the number and activity of mast cells within the airway epithelium and smooth muscle.^15^

**Figure 1:**
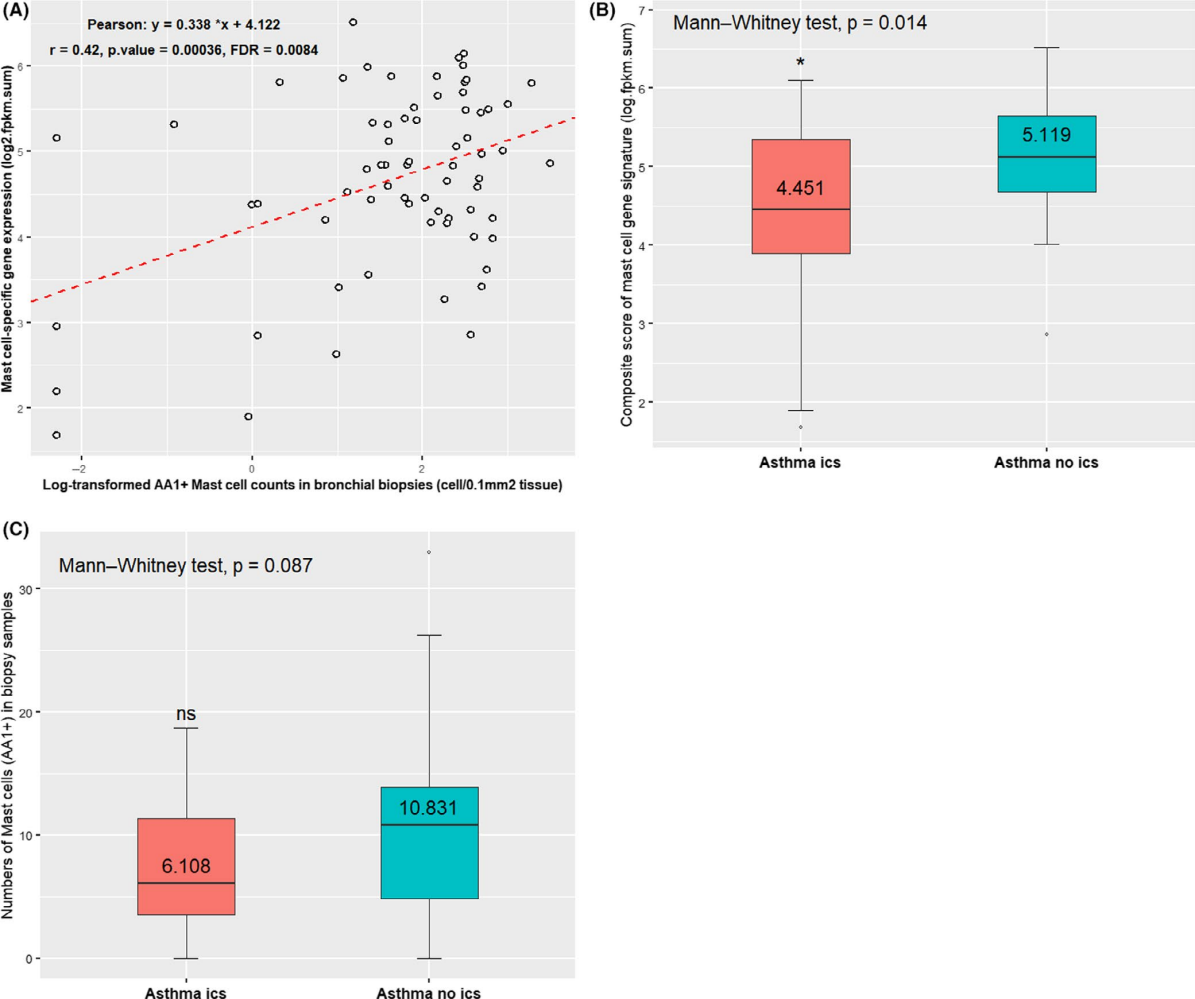
Relationship between mast cell gene signature and measurements of their cell counts in biopsy samples. A, The mast cell signature is positively correlated with the tryptase‐containing mast cells (AA1+) in biopsy samples from 69 asthma patients. B, The composite score of the mast cell gene signature is significantly decreased in asthma patients receiving ICS than in patients without ICS. C, The numbers of mast cells (AA1+) are reduced in asthma patients with ICS treatment compared to patients without ICS

Overall, our approach, calculating a composite score for mast cell‐specific genes, captures the transcript abundance of the mast cells and thus (a) can accurately quantify mast cell gene expression in bulk RNA‐seq and (b) may reflect both the number and activity of mast cells in the airway epithelium, complementing the IHC‐based mast cell counts with information about behaviour of cells in biopsy sections.

## Data Availability

The data that support the findings of this study are openly available in [repository name e.g “figshare”] at http://doi.org/[doi], reference number [reference number].
